# Amyloidogenesis via interfacial shear in a containerless biochemical reactor aboard the International Space Station

**DOI:** 10.1038/s41526-022-00227-2

**Published:** 2022-09-20

**Authors:** Patrick McMackin, Joe Adam, Shannon Griffin, Amir Hirsa

**Affiliations:** 1 Mechanical, Aerospace, and Nuclear Engineering, Rensselaer Polytechnic Institute, 110 8th St, Troy, 12180 NY USA; 2 Department of Biological Sciences, Rensselaer Polytechnic Institute, 110 8th St, Troy, 12180 NY USA; 3 Chemical and Biological Engineering, Rensselaer Polytechnic Institute, 110 8th St, Troy, 12180 NY USA

**Keywords:** Biological physics, Fluid dynamics

## Abstract

Fluid interfaces significantly influence the dynamics of protein solutions, effects that can be isolated by performing experiments in microgravity, greatly reducing the amount of solid boundaries present, allowing air-liquid interfaces to become dominant. This investigation examined the effects of protein concentration on interfacial shear-induced fibrillization of insulin in microgravity within a containerless biochemical reactor, the ring-sheared drop (RSD), aboard the international space station (ISS). Human insulin was used as a model amyloidogenic protein for studying protein kinetics with applications to in situ pharmaceutical production, tissue engineering, and diseases such as Alzheimer’s, Parkinson’s, infectious prions, and type 2 diabetes. Experiments investigated three main stages of amyloidogenesis: nucleation studied by seeding native solutions with fibril aggregates, fibrillization quantified using intrinsic fibrillization rate after fitting measured solution intensity to a sigmoidal function, and gelation observed by detection of solidification fronts. Results demonstrated that in surface-dominated amyloidogenic protein solutions: seeding with fibrils induces fibrillization of native protein, intrinsic fibrillization rate is independent of concentration, and that there is a minimum fibril concentration for gelation with gelation rate and rapidity of onset increasing monotonically with increasing protein concentration. These findings matched well with results of previous studies within ground-based analogs.

## Introduction

Protein biology in spaceflight is a field of research that has been expanding along with the advancement of space exploration and human habitation in altered gravity^1–5^. Studying the biophysical and fluid dynamic behavior of liquid protein solutions in microgravity can provide insight into fundamental physical phenomena in space, as well as within biochemical systems on Earth. In space, the absence of gravity increases the prominence of the air-liquid interface, material properties such as surface tension, surface viscosities, and molecular adsorption becoming even more impactful to the behavior of a liquid system^1,2^. On Earth many biochemical systems exist where fluid interfaces have major effects of key importance, including environmental surfactant layers^6^, industrial bioprocessing^7^, and physiological tissue surfaces^8^ within the body. Such systems with fluid interfaces, both in space and on Earth, can exhibit unique alterations in fluid and protein behavior due to protein adsorption^9^, fibrillization^10^, biopolymer dynamics^11^, and gelation^12^, all dependent upon the fluid system’s geometry and the specific type of proteins present.

Human insulin was selected as the model protein for this study based on two main points of significance: relevance to studies in microgravity and relevance to protein biophysics with interfacial hydrodynamics. Insulin’s relevance to spaceflight originates from the protein’s history of kinetics and crystallization in microgravity^1,13–15^, medical applications to diabetogenic effects in human spaceflight^2,5,16–18^, and application as a model pharmaceutical^7,19,20^ for studies of protein stability and in situ resource utilization in spaceflight. From a biophysics and fluid physics perspective, insulin displays rich bulk hydrodynamic^21–23^, interfacial^24–28^, and protein kinetics^29–33^ behavior. Moreover, insulin is an amyloidogenic protein that can undergo a fibrillization process, termed amyloidogenesis, to produce amyloid fibrils which possess a durable beta-cross quaternary protein structure^10,32,34^. Many other amyloidogenic proteins besides insulin exist^34^, some of which are functional amyloids^35–37^ which support natural biological functions while others are related to disease, such as the beta amyloid^38–41^ and tau^42,43^ proteins of Alzheimer’s disease, alpha-synuclein^44,45^ of Parkinson’s disease, infectious prion^46,47^ proteins, and the islet protein^48–50^ involved in type 2 diabetes. Comparatively, insulin is a relatively safe model amyloidogenic protein for space studies, as dangerous proteins such as infectious prions are not allowed on the ISS due to safety regulations^51^. Overall, insulin is a multifaceted model for studying protein interfacial rheology and kinetics with relevance to biophysics, fluid physics, medicine, and spaceflight.

The process of amyloidogenesis applicable to human insulin and other amyloidogenic proteins, progresses in three key biophysical stages: nucleation, fibrillization, and gelation^10,29,32^. Nucleation is the joining of two monomers, a molecular association that often changes secondary and tertiary protein structure, to form a pre-fibril aggregate, or nucleate, with quaternary structure that can accept additional monomeric subunits, seeding the system with starting points for fibrillization^10,34^. Fibrillization, or more specifically elongation, is the addition of a monomer to an existing fibril, a polymerization process which lengthens protein polymers from pre-fibril aggregates, to fibrils, to fibers, with the distribution of fibril size changing as the process progresses^10,12^. Gelation is the linking of protein fibers to form a polymer network, a structure filled with solvent which maintains a defined shape, a process that can occur concurrently with fibrillization if sufficiently large fibril sizes are present^12,29,32^. These defining protein kinetic processes of amyloidogenesis are governed by a system’s dynamic microenvironment, and lead to changes in protein mechanical properties^32,52–55^, cytotoxic effects in amyloid diseases^39,40,42,45,46,48–50,55–57^, and formation of intricate nanostructure^58,59^, potentially adaptable to tissue engineering.

The chemical and thermodynamic state of a system governs protein kinetics, biophysical processes of the constituent molecules progressing toward a point of lower free energy^10,12^. Fluid transport and associated hydrodynamic stresses influence the thermodynamic state of amyloid systems, both bulk shear flow^21–23,60–62^ and air-liquid interface activity^24–28,63–67^ affecting the number of molecular collisions, with more frequent collisions increasing the probability for interactions including nucleation, fibrillization, and gelation. Geometries with fluid interfaces are well-suited to the study of physiological systems as most interfaces within the body are fluidic in nature, flow of cerebrospinal fluid (CSF) within the brain being of specific importance for many neurodegenerative diseases^8,62,68–72^. Furthermore, brain structure and CSF have been observed to undergo alterations due to spaceflight^73–78^, making the study of such systems imperative to long-term space habitation. Along with these fluid effects, protein kinetics are also affected by the quantity of protein present, which can alter molecular dynamics and the overall interactions with bulk fluid and fluid interfaces.

**Fig. 1:**
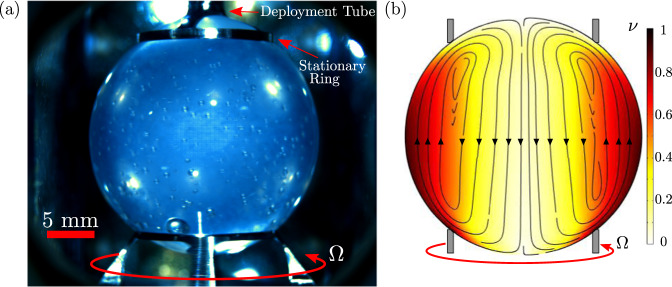
The ring-sheared drop (RSD) with the bottom ring rotating. **a** Image of the ring-sheared drop aboard the ISS showing a pre-sheared 8 mg/mL human insulin solution spinning at 30 rpm. **b** Axisymmetric computation of primary (color map of normalized azimuthal velocity, *ν*) and secondary (black arrowed streamlines in the azimuthal plane) flow of a Newtonian fluid in the RSD sheared at 30 rpm.

Microgravity provides a unique environment for the experimental study of systems with solely fluid interfaces, the dominance of free surfaces in the absence of gravity facilitating the removal of solid boundaries which often introduce nucleation sites and unintended wall effects. The ring-sheared drop (RSD) is a containerless surface tension-contained microgravity biochemical reactor consisting of a 2.54 cm diameter drop pinned between two rings, one stationary and one shearing, that transfer interfacial shear to the bulk fluid by surface shear viscosity and mix the liquid using inertial flow with secondary motion (Fig. 1)^79–83^. The RSD was first deployed to the international space station (ISS) in the Fall of 2019 and again for a second operations campaign in the Summer and Fall of 2021. A ground-based preliminary study^28^ has been performed with human insulin in the Earth analog of the RSD, the knife-edge viscometer (KEV)^84^, an apparatus which requires a glass dish for containment under gravity yet, like the RSD, produces shear flow using surface shear viscosity and mixes via secondary flow. The present investigation examined human insulin within the RSD aboard the ISS to measure the effects of steady interfacial shear on the amyloidogenesis processes of nucleation, fibrillization, and gelation in a microgravity, air-liquid interface dominated system. Specifically, the hypothesis tested was that if protein solutions in microgravity are seeded with fibrils and subject to steady interfacial shear, then amyloidogenesis will occur with the extent of gelation depending on total protein concentration.

## Results

### Pre-sheared fibrillization

**Fig. 2:**
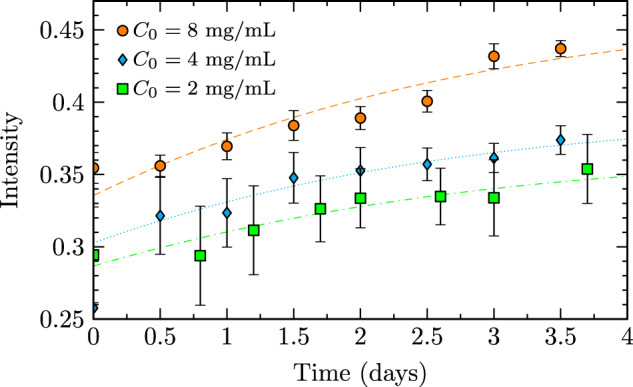
Intensity of scattered light verses time for pre-sheared insulin cases. ± 1 standard deviation error bars represent measurement uncertainty and dashed lines represent sigmoidal fits to a theoretical fibrillization function (Eq. 1) presented in the Methods section.

Three pre-sheared trials were performed in this investigation at total protein concentrations of 2, 4, and 8 mg/mL, each subject to steady interfacial shear at 30 rpm for 3.5 days. Image data was captured every 0.5 days for characterization of fibrillization kinetics. Measured intensity of image data was used to construct fibrillization curves of intensity versus time, as depicted in Fig. 2. Measured intensity increased monotonically with time for all samples, indicative of the presence of fibrils with larger aggregates producing increased scattering and higher image intensity. Curves in Fig. 2 represent nonlinear least-square fits to a theoretical sigmoidal fibrillization function (Eq. 1) that align well with experimentally measured data.

### Pre-sheared fibrillization kinetics

**Fig. 3:**
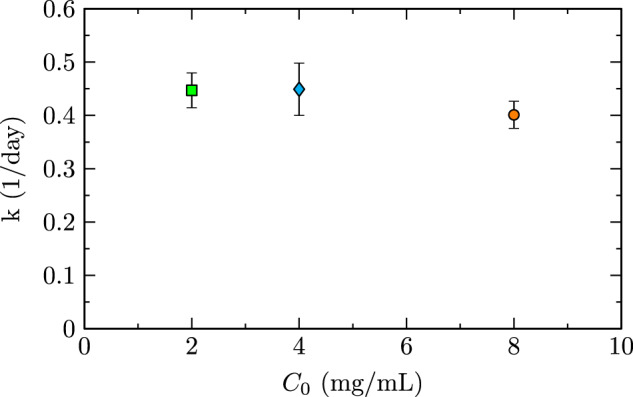
Intrinsic fibrillization rate *k* versus protein concentration *C*_0_. Values of *k* were determined by fitting to a fibrillization model (Eq. 1). Error bars represent fit uncertainty.

Curves in Fig. 2 were quantified by fitting measured data to a theoretical fibrillization function with empirical parameters of biophysical relevance (Eq. 1). Importantly, this equation does not directly model fibrillization when applied to intensity data (Fig. 2), as this theoretical function typically applies to fibril content measured using spectroscopy as opposed to solution intensity. The first adjustment required for intensity fitting was a horizontal offset to account for pre-shearing of these fibrillization trials, as these trials did not begin as completely native solutions. The second adjustment was that the fitting parameter typically describing total protein concentration, *I*_0_, instead described the intensity of a fully monomeric protein solution. This value was used to improve fits by providing a measureable vertical offset due to background monomer intensity. Fit rms error (Table 1 column 3) was < 3% for all cases. Larger fit errors (8 mg/mL) resulted from measurement accuracy reduction due to high sample intensity, which can slightly effect curve shape due to near-upper limit sensor values. The altered application of this equation to optical intensity as opposed to spectroscopic fibril content remained suitable for the study of fibrillization kinetics with a focus on intrinsic fibrillization rate. The fit-determined native intensity values, *I*_0_, are displayed in Table 1. Furthermore, sigmoidal fits allowed for relation between intrinsic fibrillization rate and total protein concentration (Fig. 3). Intrinsic rate was shown to be independent of concentration and thus, the time scale of fibrillization not dependant on protein concentration, a result consistent with the ground study^28^.

**Table 1:** Sigmoidal fit (Eq. 1) parameters of native intensity (*I*_0_) and normalized fit root-means-square (rms) error.

*C*_0_ (mg/mL)	*I*_0_	rms Error (%)
4	0.49 ± 0.03	1.3
2	0.33 ± 0.02	2.1
8	0.88 ± 0.02	2.7

### Seeded fibrillization

**Fig. 4:**
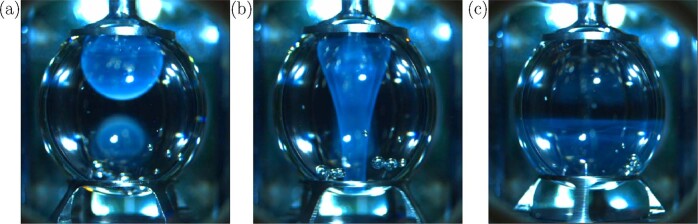
Images of the native 2 mg/mL insulin case being seeded at 5.6 days with 0.4 mL of insulin fibrils. Panels show the injected volume (**a**) before and (**b**, **c**) after restarting ring rotation. Mixing of the turbid fibrillized volume allowed for visualization of the RSD's secondary flow (Fig. 1b and Supplement1.mp4).

**Fig. 5:**
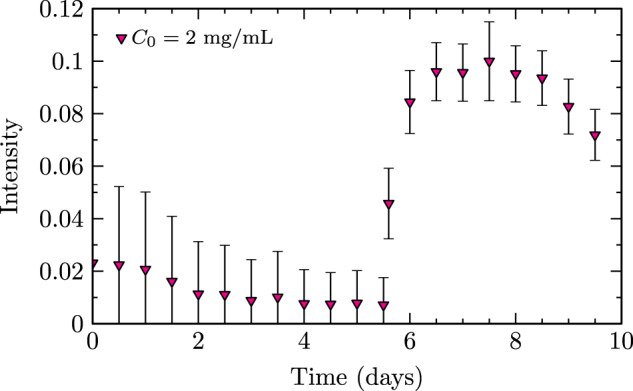
Intensity of scattered light versus time for the 2 mg/mL native insulin case with seeding by insulin fibrils at 5.6 days. ±1 standard deviation error bars represent measurement uncertainty.

The seeding trial occurred during measurement of a native (fully monomeric) 2 mg/mL sample. After 5.6 days an additional injection of 0.4 mL of fluid from the deployment tube was made to account for evaporative losses and return the drop to a spherical shape. Serendipitously, this injection provided a means to measure the effect of seeding with fibril aggregates. The inside of the deployment tube, where remnants of the injection volume had resided for 5.5 days, was a rough unfinished stainless steel surface that provided ample nucleation sites for fibrils to form, which were subsequently transported into the bulk of the RSD during the seeding injection. The turbidity of this fibril seeding injection was readily observable, and upon shear restart, the resulting mixing within the drop allowed for visualization of the RSD’s secondary inertial flow as displayed in Fig. 4 (see also Supplement1.mp4). Following the initial increase in intensity due to added fibril content, intensity continued to increase, as the solution had begun fibrillizing due to the previous seeding event (Fig. 5). The slight decrease in measured intensity observable at 9.0 and 9.5 days was unexpected and may be due to fibril adsorption to liquid-solid interfaces of the rings^27^, which could have drawn particles into areas of the flow occluded from the camera’s field of view.

### Pre-sheared gelation

**Fig. 6:**
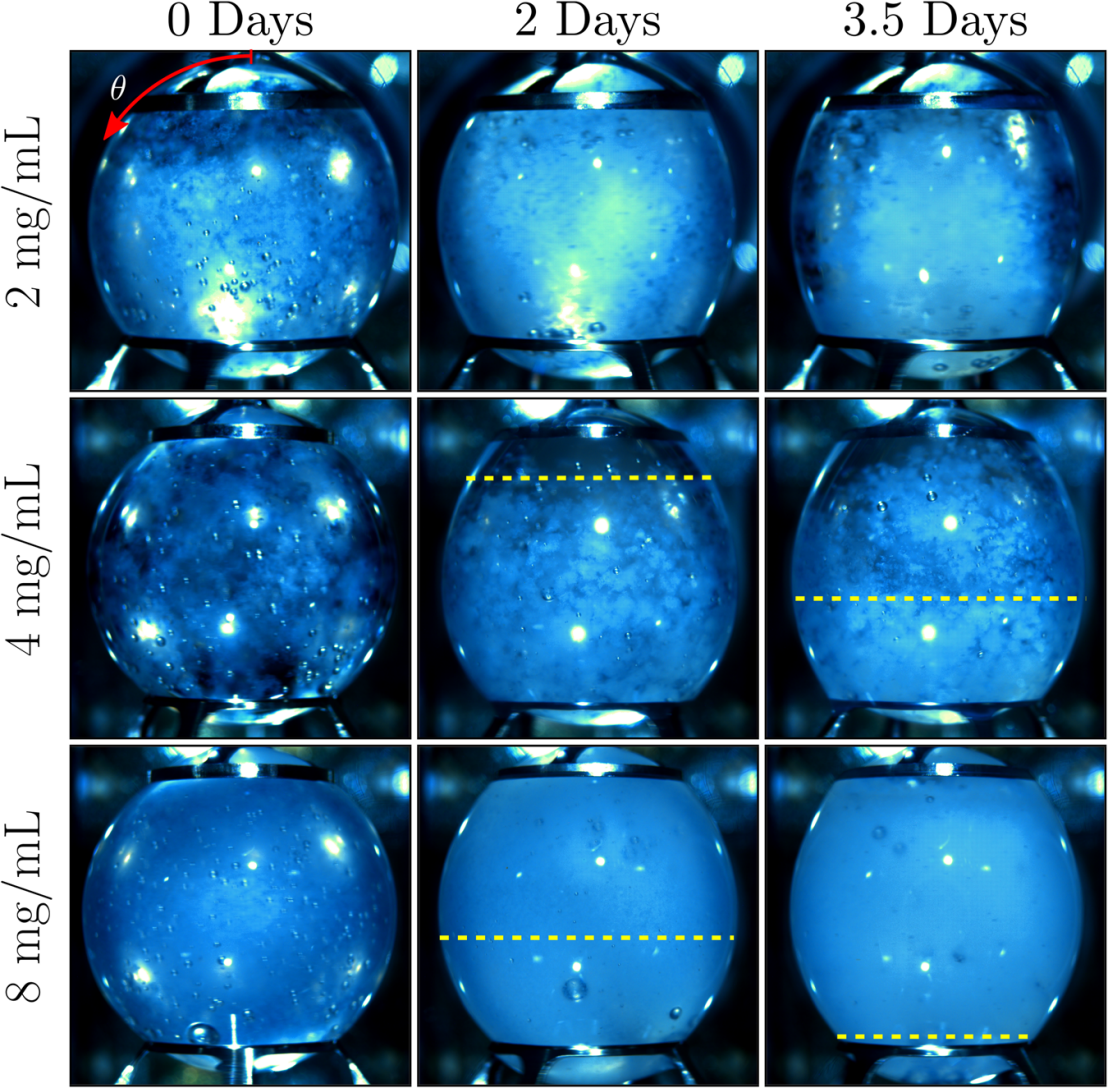
Images of gelation front progression in pre-sheared insulin cases. If present, southward-moving gelation fronts are indicated by yellow dashed lines.

**Fig. 7:**
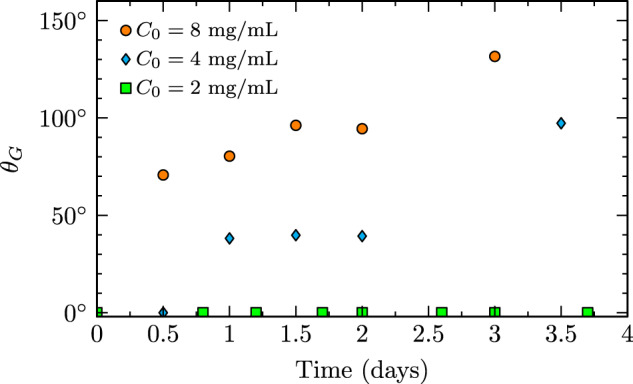
Gelation front polar position, *θ*_*G*_, vs time for pre-sheared insulin cases. This data was extracted using the videos from which Fig. 6 was generated.

Protein gelation was observed by the presence of a turbid stationary solidification front moving southward from the stationary ring. Figure 6 depicts the progression of this gelation front with time. The 2 mg/mL sample did not reach a sufficiently high fibril concentration in order to produce gelation. This requirement of a minimum fibril concentration for gelation is consistent with previous ground-based studies^28,29^. Figure 7 quantifies the progression of these gelation fronts with time in terms of a gelation front polar angle, *θ*_*G*_. Both the rate of progression and rapidity of onset of gelation increase with increasing protein concentration. To further this observation, the 8 mg/mL case had transitioned entirely to a linked polymer gel by the trial’s conclusion, preventing liquid extraction and remaining affixed to the rings even after test cell removal.

## Discussion

Fluid interfaces produce significant effects on the biophysics of protein solutions, defining the microenvironment and energetic landscape^10,12^ through processes such as molecular adsorption^9^ and imparted forces such as interfacial shear^7^ affecting biological behaviors including the fibril dynamics^10,11^ and gelation^12^ of proteins. This investigation was the space continuation of an Earth-based study^28^, this work studying amyloidogenesis, production of amyloid fibrils and plaques from native amyloidogenic proteins, of human insulin in an air-liquid interface dominated biochemical reactor in microgravity. This fluid system, the RSD aboard the ISS, offered a platform for studying both the effects of fluid interfaces and microgravity on protein fibrillization. Amyloidogenesis of human insulin was used as a model biophysical system due to its applicability to biotechnology, physiology, medicine, and spaceflight.

Three stages of amyloidogenesis were quantified in this study, including seeding, fibrillization, and gelation of insulin. A native solution of insulin serendipitously seeded with insulin fibrils (Fig. 4) displayed an earlier onset of fibrillization (Fig. 5). This accelerated onset of fibrillization due to seeding is applicable to diseases such as infectious prions^46,47^ or biotechnological processes that introduce a portion of fibrils to a native solution and in turn promote fibrillization of the native solution. Fibrillization experiments with pre-sheared insulin samples (Fig. 2) displayed the induction of amyloidogenesis via steady axisymmetric interfacial shear, with intrinsic fibrillization rates (Fig. 3) independent of total protein concentration. This concentration independence matches results of the ground study^28^ and indicates that forces imposed at the fluid boundary lead to changes in the protein microenvironment that govern the timescale of fibrillization. The average value of this rate constant, 0.43 ± 0.07 1/days, matches the expected value (0.47 1/days) obtained by a logarithmic extrapolation using ${Re}$ of fibrillization rates from Fig. 4a of the ground study^28^. Gelation was observed (Fig. 6) in the form of gelation fronts, only in cases with sufficiently high protein fibril concentration to from crosslinked polymer networks, and the rapidity of gelation onset and rate of gelation (Fig. 7) increased monotonically with increasing protein concentration.

This study marks the first successful use of the RSD fluid apparatus (Fig. 1) with protein solutions in microgravity aboard the ISS. Hardware and biological samples were transported to the ISS and installed in the MSG without fault. During operation, this device successfully deployed, pinned, steadily sheared, and extracted fluid drops of protein solutions, demonstrating performance of a novel method for producing air-liquid interface dominated systems in microgravity. Optics, deployment, shearing, and complete operation of the apparatus were successfully performed using real-time remote control from the ground. With minimal impact of solid boundaries, sole components being the thin contact rings used to transmit interfacial shear, this device is well-suited to the study of interfacial phenomena and the dynamics of fluid interfaces.

Results of this investigation center on the three main aspects of amyloidogenesis: nucleation, fibrillization, and gelation. Seeding of protein solutions in microgravity was shown to promote earlier onset of fibrillization by bolstering the nucleation process. Fibrillization was demonstrated to be promoted by interfacial shear, with the intrinsic rate of fibrillization being independent of protein concentration. Gelation was found to require a critical concentration of protein fibrils with gelation onset and rate becoming more rapid with increasing protein concentration. Furthermore, findings presented here demonstrate the successful performance of the microgravity biochemical reactor, the RSD, utilized in this investigation. A multitude of future space investigations exist that could make use of an interfacial biochemical reactor such as the RSD, including studies on drop rheology, interface creation and substrate interaction, different interfacial flow regimes, and microbial biofluids. Drop rheology in space is applicable to fields ranging from fundamental changes in fluid behavior^85–88^ to the study of planetary bodies^89–91^ and their material properties. Studies of interface creation and substrate interaction can be used to describe fundamental contact line dynamics^92–100^, 3D printing^87,101^, and combustion^102,103^ in microgravity. Use of select interfacial flow regimes such as steady, pulsatile, or oscillatory flows, allows fluid devices to mimic the effects of flow in physiological systems such as the gastrointestinal^104,105^, glymphatic^73–78^, circulatory^106,107^, or respiratory^108,109^ systems and the effects of spaceflight on these systems and the cells^110,111^ within. The behavior of microbial biofluids is important for understanding the effects of spaceflight on microbiology^112–121^ and potential applications to pharmaceutical production and bio-engineering^4,117,122,123^ in support of space exploration. Future investigations of interfacial hydrodynamics in microgravity could offer insight into fluid systems that better facilitate spaceflight.

## Methods

### RSD

The RSD consists of a 2.54 cm diameter spherical liquid drop pinned between two thin titanium contact rings (Fig. 1). The top ring is connected via four prongs to a 10-gauge stainless-steel deployment tube used to grow the liquid drop. The lower ring rotates to produce interfacial shearing of the drop, shear being transmitted to the bulk by means of surface shear viscosity and mixing occurring due to secondary flow^79–83^. The RSD was conceived in 2013, and the hardware was developed and launched to the ISS on SpaceX CRS-18 July 2019 after a series of parabolic flights. Following these engineering missions, the science mission (including biological samples and hardware modifications presented in this study) was launched on Cygnus NG-16 in August 2021, operations being performed in the following months concluding in December 2021. The RSD hardware was operated within the Microgravity Science Glovebox (MSG), located in the Destiny module of the ISS, providing 3 levels of containment (test cell, MSG airflow, and MSG wall) between the astronauts and protein samples. Due to the low 1.6 pH, samples were classified as a hazard response level 2 material (HRL), which necessitated at least 3 levels of containment in accordance to NASA crew-safety requirements^51^.

### Protein samples

Protein samples of human insulin were prepared by dissolving lyophilized recombinant human insulin (Sigma-Aldrich, 91077C) in a 0.1 M NaCl 1.6 pH buffer solution (deionized water, pH cycled with HCL and NaOH) to pharmaceutical-relevant^124,125^ concentrations of 2, 4 and 8 mg/mL. The low pH of the buffer allowed for contamination resistance in addition to pH control. Each sample was pre-sheared for 1 day at a Reynolds number of 6000 in a deep-channel surface viscometer^26,126^ which produced partial fibrillization that allowed for earlier onset of fibrillization during operations in space. Additionally, a native (fully un-fibrillized monomeric, or dimeric at 1.6 pH) 2 mg/mL sample was also prepared, ultimately used in a serendipitous seeding trial. After preparation, all samples were degassed under a 710 mmHg vacuum for 0.5 days to minimize air bubbles and subsequently frozen at −20 ^∘^C before transportation and launch to the ISS. On orbit, sample syringes were thawed 1 day at ambient temperature before installation in the RSD hardware. After each experimental trial, each protein solution was withdrawn back from the drop into the syringe and placed in cold stowage (4 ^∘^C) until returning to Earth. Each sample syringe had two controls, a flight control which accompanied samples to the ISS and a ground control that remained on Earth. Both controls underwent no shear experimentation and were identically degassed, frozen, thawed and subsequently refrigerated.

### Experimental trials

Experimental trials began after crew members installed the 12 mL sample syringe and test cell (test cells containing the RSD’s pinning rings and deployment tube) into the experimental hardware and sealed the MSG. Drop deployment followed installation by 0.5 days to dissipate any static charge accumulated during installation and to place deployment during crew sleep, avoiding deleterious accelerations. Drops were deployed at a rate of 10 mL/min in two stages to the total volume of 8.58 mL (volume of a 2.54 cm diameter sphere). Steady shear commenced after deployment with the lower ring rotating at 30 rpm, corresponding to a Reynolds number of 180 (where Re = Ω*a*^3^/*ν*, where Ω is ring rotation rate, a is ring radius, and *ν* is the kinematic viscosity of water). Wide-field image data (example in Fig. 1a) of steadily-sheared drops was collected every 0.5 days, with pre-sheared trials shearing for 3.5 days and the native trial shearing for 9.5 days with a midpoint seeding injection of 0.4 mL of insulin fibrils after 5.6 days. LED light modules used for illumination were deactivated outside of sampling to maintain ambient conditions within the test cell.

### Protein amyloidogenesis

Protein amyloidogenesis was quantified using the measured intensity of light from a drop’s bulk fluid in both the native seeding trial and the pre-sheared fibrillization trials. As fibrillization proceeded the size of particles within a solution increased leading to increased scattering of light and visible increases in turbidity. While such optical methods of measurement lag behind spectroscopic measurements of monomer extinction^23^ (as sufficiently small fibrils and pre-fibril aggregates will not scatter light), trends in fibrillization remain observable. Measured intensity was defined as the average of normalized red, green, and blue intensity values within an interrogation area centered on the drop, calculated using a MATLAB script summing over the 120 frames (30 fps) recorded at each time point. Measurement uncertainty was defined as the standard deviation of these intensity values within the interrogation window over each of the 120 frames of the video camera. This average intensity was then normalized to produce the measured intensity, *I*, which ranged between 0 and 1 corresponding to no detected light and a fully saturated camera sensor. Furthermore, optical measurement was also utilized for the quantification of the final stage of amyloidogenesis, protein gelation. As a protein solution transitioned from a liquid suspension of fibers to a linked network of fibers, a noticeable change occurred in the optical properties of regions undergoing this phase transition. Differences in turbidity, highly turbid unmoving regions indicative of gel, allowed for the measurement of gelation progression using optical tracking of gelation fronts.

### Model fit

A three-parameter sigmoidal function was utilized to obtain *I*_*f*_(*t*), representing a solution’s intensity based on a specific fibril content as a function of time:

1 $${I}_{f}(t)={I}_{0}\left(\frac{1}{1+{e}^{k({t}_{h}-t)}}-\frac{1}{1+{e}^{k\cdot {t}_{h}}}\right).$$

This three-parameter function originated from protein fibrillization theory and contains constants that are of relevance to biophysical properties^10,25,29^. *I*_0_ is the initial intensity describing a fully monomeric protein solution, *t*_*h*_ is the time (days) required to reach a half fibrillized solution, and *k* is the intrinsic rate coefficient (1/days) that depends on both the processes of nucleation and fibril elongation. Measured intensity values of the pre-sheared trials were fit to Eq. (1) using a nonlinear least-squares MATLAB algorithm to obtain these biophysical parameters as functions of protein concentration.

### Reporting summary

Further information on research design is available in the Nature Research Reporting Summary linked to this article.

## Supplementary information

Mixing of injected fibrils

Reporting Summary

## Data Availability

The data collected during this study is available from the corresponding authors upon reasonable request.

## Code availability

The code written during this study is available from the corresponding authors upon reasonable request.
